# Correction: Multi-stimuli programmable FRET based RGB absorbing antennae towards ratiometric temperature, pH and multiple metal ion sensing

**DOI:** 10.1039/d3sc90045b

**Published:** 2023-03-09

**Authors:** Kavita Rani, Sanchita Sengupta

**Affiliations:** a Department of Chemical Sciences, Indian Institute of Science Education and Research (IISER) Mohali Punjab-140306 India sanchita@iisermohali.ac.in

## Abstract

Correction for ‘Multi-stimuli programmable FRET based RGB absorbing antennae towards ratiometric temperature, pH and multiple metal ion sensing’ by Kavita Rani *et al.*, *Chem. Sci.*, 2021, **12**, 15533–15542, https://doi.org/10.1039/D1SC05112A.

The authors regret that the unit of the temperature sensitivity was given incorrectly in the Abstract, the section on Ratiometric temperature sensing, and the Conclusion of the article. The unit of the temperature sensitivity should be %°C^−1^, rather than %°C.

The Royal Society of Chemistry apologises for these errors and any consequent inconvenience to authors and readers.

## Supplementary Material

